# Predictive value of plasma sICAM-1 and sP-Selectins in the risk of death in patients with acute respiratory distress syndrome

**DOI:** 10.5937/jomb0-45340

**Published:** 2024-04-23

**Authors:** Pan Jing, Chaomin Wu, Chunling Du, Lei Zhou, Liang Gu

**Affiliations:** 1 Qingpu Branch of Zhongshan Hospital Affiliated to Fudan University, Department of Pulmonary and Critical Care Medicine, Shanghai City, China

**Keywords:** sICAM-1, sP-Selectins, acute respiratory distress syndrome, risk of death, sICAM-1, sP-selektini, akutni respiratorni distres sindrom, rizik od smrti

## Abstract

**Background:**

To evaluate the predictive value of sICAM-1 and sP-Selectins in the risk of death in a prospective cohort of adult acute respiratory distress syndrome (ARDS).

**Methods:**

Adult ARDS patients were included. Plasma sICAM-1, sP-Selectins, and inflammatory cytokines (TNF-α, IL-1b, IL-6, IL-8, and IL-17A) were detected in ARDS subjects. The correlation between different factors and the potential of sICAM-1 and sP-Selectins as endothelial markers to predict the risk of deathfrom ARDS was analyzed.

## Abstract (sr)

**Uvod:**

Cilj je bio da se proceni prediktivna vrednost sICAM-1 i sP-selektina u riziku od smrti u prospektivnoj kohorti akutnog respiratornog distres sindroma odraslih (ARDS).

**Metode:**

Uključeni su odrasli pacijenti sa ARDS-om. Plazma sICAM-1, sP-selektini i inflamatorni citokini (TNF-α, IL-1b, IL-6, IL-8 i IL-17A) otkriveni su kod ispitanika sa ARDS-om. Analizirana je korelacija između različitih faktora i potencijala sICAM-1 i sP-selektina kao endotelnih markera za predviđanje rizika od smrti od ARDS-a.

**Rezultati:**

sICAM-1 i sP-selektini u plazmi bili su viši kod pacijenata sa ARDS-om i viši kod onih koji nisu perživeli ARDS. sICAM-1 je bio značajno povezan sa TNF-α, IL-6 i IL-8, a sP-selektini su takođe značajno korelirali sa IL-6 i IL-8. Iznad kritičnih vrednosti od 245,5 ng/mL (sICAM-1) i 482,5 ng/mL (sP-selektini), smrtnost populacije ARDS-a se povećala. Visoki sICAM-1 i sP-selektini u plazmi na prijemu u intenzivnu terapiju bili su nezavisni prediktori smrti pacijenata sa ARDS-om.

**Zaključak:**

sICAM-1 i sP-Selektini mogu predvideti rizik od smrti kod kritično bolesnih pacijenata sa ARDS-om.

## Introduction

According to the Berlin definition, acute respiratory distress syndrome (ARDS) is an acute, diffuse, and inflammatory lung injury [1]. An international multicenter epidemiological study published in 2016, combining reports from 459 intensive care unit (ICU) patients from 50 countries, showed that among 29,144 ICU patients, 10.4% met the ARDS criteria, and the in-hospital death rates for mild, moderate, and severe ARDS were 34.9%, 40.3%, and 46.1%, respectively [2]. According to the study of Madotto Fabiana et al. [3], the death rate of patients with ARDS can reach 31% even if they get rapid remission. At present, the treatment of ARDS is mainly based on low tidal volume lung protection ventilation, limiting fluid input, and other comprehensive treatments, and there is no effective treatment drug. Prevention is the key to reduce the death of ARDS. Therefore, the study of biomarkers closely related to ARDS has high clinical value for the early diagnosis, treatment, and prevention of ARDS.

ARDS can cause pulmonary endothelium-related injury, including diffuse endothelial injury, activation of the coagulation system, and increased capillary permeability. Lung endothelial cells are metabolically active continuous monolayer squamous endothelial cells that mediate key processes involved in lung homeostasis [4]. A breakdown in the integrity of the endothelial barrier is characteristic of many inflammatory states. Adhesion molecules are glycoproteins expressed on the cell surface that mediate contactbetween two cells (homotypic and heterotypic interactions) or between cells and extracellular matrix.

Intercellular Adhesion Molecule-1 (ICAM-1) is divided into soluble ICAM-1 (sICAM-1) and membrane ICAM-1 (mICAM-1), and ICAM-1 exists in the form of sICAM-1 outside the cell [5]. sICAM-1 is a transmembrane glycoprotein related to the structure of the immunoglobulin surface gene family, which is specifically involved in the transport of inflammatory cells, leukocyte effector function, adhesion of antigen-presenting cells to T lymphocytes, microbial pathogenesis, and signaling pathway through external-internal signal transduction events [6] [7]. Early studies on sICAM-1 focused on cardiovascular diseases such as atherosclerosis [8], chronic heart failure [9], and coronary heart disease [10]. sICAM-1 has been reported to be associated with disease progression and aggressiveness, such as thyroid papillary tumor [11], melanoma [12], and glioblastoma [13]. In recent years, sICAM-1 is associated with respiratory diseases. Endothelial PAS domain containing protein-1 promotes hypoxic pulmonary hypertension and aggravates hypoxic pulmonary hypertension by mediating the activation of endothelial ICAM-1 [14]. Targeting ICAM-1 can reduce rhinovirus-induced exacerbation of chronic obstructive pulmonary disease [15]. sICAM-1 and selectin regulation by oxidative stress and NF-B pathway aggravate lung injury [16].

P-selectin (CD62) belongs to calcium-dependent cell adhesion molecules that mediate specific reactions among endothelial cells, white blood cells, and platelets [17]. P-selectin can exist in plasma in soluble form, and soluble P-selectin (sP-selectin) is mainly derived from vascular endothelial cells [18]. It has been reported that inhibition of P-selectin can reduce severe acute lung injury in immunocompromised mice [19]. Moreover, an increase in sP-selectin levels is correlated with the severity of lung disease injury caused by COVID-19 [20].

In summary, clinical and laboratory evidence suggests that vascular endothelial injury is crucial in lung diseases. Most studies on biomarkers and ARDS have taken a single-pathway approach, although a single mechanism is unlikely to predict the outcome of complex syndromes such as ARDS. In this cohort of ARDS patients, the predictive value of two endothelial cell biomarkers (sICAM-1 and sP-selectin) at 60 days of death was determined, as well as the association with inflammatory factors (TNF-α, IL-1β, IL-6, IL-8, and IL-17A).

## Materials and methods

### Subjects

All adult patients admitted to the intensive care unit of Qingpu Branch of Zhongshan Hospital Affiliated to Fudan University for ARDS according to the Berlin definition [1] between September 2019 and December 2022 were included. Exclusion criteria: (1) < 18 years of age; (2) Death within 24 h of admission; (3) follow-up records were not available for any reason other than death. The treatment of ARDS follows the UKguidelines for the management of adult patients with ARDS and incorporates the conditions of hospital facilities. The guidelines describe advanced therapeutic interventions, including conservative fluid management strategies; low tidal volume ventilation, mechanical ventilation with high positive end-expiratory pressure (PEEP), and neuromuscular blockers; extracorporeal membrane oxygenation is recommended as an adjoint to protective mechanical ventilation in critically ill patients with ARDS. NO inhalation is not supported.

A total of 113 patients with ARDS were enrolled, ranging in age from 23 to 85 years, with a median age of 57 years. During hospitalization, 78 patients were survivors and 35 were non-survivors. At the same time, 36 age-matched healthy subjects who came to the hospital for health examination were selected. This study was approved by the Institutional Review Board of Qingpu Branch of Zhongshan Hospital Affiliated to Fudan University. Under the Committee's guidelines, written informed consent was obtained from all patients or families.

### General clinical data

Clinical data were extracted from a data management system in the hospital. In addition to basic demographic data (gender, age, height, and weight), ICU admission scores (APACHE II, SAPS II, SOFA scores) and severity of ARDS (mild, moderate, severe) were assessed according to the Berlin definition [1].

As the main clinical causes of ARDS [21], pneumonia, sepsis, immune deficiency, "acute to chronic" (i.e., patients with existing chronic lung disease with acute exacerbation), and severe trauma were distinguished. Parameters of pulmonary gas exchange and mechanical ventilation, such as peak inspiratory pressure (Ppeak), mean airway pressure (Pmean), PEEP, PaO_2_/FiO_2_ ratio, oxygen index, and pulmonary compliance, were also assessed at ICU admission. Extracorporeal pulmonary assistance device (ECmos) use and medication use were recorded, including steroids, neuromuscular blocking agents (NMBA), and vasodilators.In addition, the duration of mechanical ventilation, length of ICU stay, and 60-day allcause death were recorded to characterize patient populations.

### Sample collection and laboratory measurement

Blood samples were collected in separation tubes (Thermo Fisher Scientific Inc., Waltham, MA, USA) from ARDS patients at the first admission (Day 1) and on days 3 and 7 after admission. Blood samples from healthy subjects were collected during a physical examination. All samples were centrifuged at 1200 × g at 4°C for 20 min and passed through a 13 mm filter (Thermo Fisher Scientific Inc.). The plasma was divided into equal parts and quickly frozen at -80°C. According to the scheme provided by the manufacturer, plasma sICAM-1 and sP-Selectin levels were measured using ICAM-1 (Soluble) Human ELISA Kit and P-Selectin (Soluble) Human ELISA Kit (R&D Systems, USA), respectively. The average concentration of each sample was determined. Commercial ELISA Kits for tumor necrosis factor-a (TNF-α), interleukin-1β, IL-6, IL-8, and IL-17A were purchased from Solaybao (Beijing, China).

### Data statistics

Discrete variables were expressed in the form of counts (percentages), and continuous variables were in the form of median (quartile range (IQR), i.e. 25%-75%). Demographic and patient characteristics, counting or categorical variables were analyzed using Person Chi-square or Fisher precision tests. For variables with skewed distribution, inter-group differences were compared by Mann-Whitney U or Kruskal-Wallis H test, and paired samples were compared by Wilcoxon Signed Rank or Friedman M test. The correlation among factors was analyzed by Spearman's correlation coefficient, and P-value was corrected by False Discovery Rate. The effectiveness of sICAM-1, sP-Selectin, and their combined measurements in predicting death was evaluated using ROC and area under the curve (AUC) measurements, and the critical value for distinguishing death was obtained by calculating Youden [22]. The factors influencing death were screened by univariate binary logistic regression. Multivariate tests of factors influencing death were performed using multiple logistic regression and Cox risk regression analysis, including variables showing statistical effects inunivariate variables. Kaplan-meier estimates were used to visualize survival differences using calculated cutoff values and to examine survival differences between patients below or above the calculated cutoff values using logarithmic rank tests. OR and 95% confidence intervals (CI) were calculated. Bilateral *P* <0.05 was considered statistically significant. Data analysis was performed using SPSS software Version 24, JMP statistical software Version9.0.1 (SAS, Cary, NC), and GraphPad PRISM Version 7 (San Diego, CA, USA).

## Results

### Patient characteristics

Critically ill patients admitted to the hospital for ARDS were treated and screened for this study. The final study population was 113 patients with ARDS. The characteristics of the population were studied by survivors and non-survivors (Table 1). Non-survivors were associated with significantly higher severity scores (SAPS II and APACHE II) at ICU admission. In addition, non-survivor patients were more likely to develop more severe ARDS and exhibit significantly lower lung compliance, longer mechanical ventilation times, and longer ICU stays. In addition, there were no significant differences in the etiology, treatment, and general information of ARDS between survivors and non-survivors (Table 1). However, plasma TNF-α, IL-1β, IL-6, IL-8, and IL-17A were significantly higher in non-survivors than in survivors (Table 1).

**Table 1:** Comparison of clinical features of ARDS patients in survivors and non-survivors. Discrete variables are expressed as numbers or percentages (%) and analyzed using Person chi-square or Fisher exact tests. Continuous variables were expressed as median (interquartile interval (IQR)) and analyzed for survivors and non-survivors using the Mann-Whitney U test. BMI: Body mass index; OI: Oxygenation index; CL: Pulmonary compliance; ECMO: Extracorporeal membrane oxygenation; NMBA: Neuromuscular blocking agents.

	All<br>(n=113)	Survivors<br>(n=78)	Non-survivors<br>(n=35)	p value
Basic characteristics				
Age (years)	57 (48–75)	56 (47–72)	59 (50–76)	0.053
Male sex, n (%)	58 (51.32)	40 (51.28)	18 (51.43)	0.989
BMI (kg/m^2^)	27 (23–33)	28 (22–35)	26 (23–32)	0.183
Severity of ARDS				
Mild, n (%)	11 (9.73)	8 (10.26)	3 (8.57)	1
Moderate, n (%)	50 (44.25)	39 (50.00)	11 (31.43)	0.066
Severe, n (%)	52 (46.02)	31 (39.74)	21 (60.00)	0.046
Severity of illness scores at ICU admission				
SAPS II	59 (52–71)	56 (49–62)	64 (54–73)	0.02
APACHE II	24 (17–32)	22 (17–26)	26 (23–35)	0.01
SOFA II	10 (8–14)	10 (8–13)	11 (9–14)	0.056
Pulmonary gas exchange and mechanical ventilation (at ICU admission)				
Ppeak (cm H_2_O)	34 (29–38)	33 (29–38)	34 (29–39)	0.452
Pmean (cm H_2_O)	22.8 (19.8–27.8)	22.3 (20.8–27)	23.1 (21.3–28)	0.504
PEEP (cm H_2_O)	16.0 (14.8–19.6)	15.9 (15–20)	17 (15–20)	0.391
Total volume/PBW (mL/kg)	6.0 (4.3–7.8)	6.2 (5.2–7.9)	5.8 (4.3–7.2)	0.286
PaO2/FiO2	152 (103–218)	160 (135–218)	141 (109–183)	0.106
OI	16.9 (11.0–28.2)	16.9 (12.0–28.3)	17.1 (11.0–28.2)	0.353
CL (mL/cm H_2_O)	26.5 (20.9–37.6)	33.1 (24.6–43.5)	25.6 (19.8–27.6)	< 0.001
Mechanical ventilation<br>(hours)	325 (172–682)	289 (165–523)	425 (354–853)	< 0.05
ICU length of stay (days)	16 (7–30.5)	14 (7–25)	20 (11–36)	< 0.05
Etiology of ARDS				
Pneumonia	48 (42.48)	30 (38.46)	18 (51.43)	0.197
Sepsis	21 (18.58)	18 (23.08)	3 (8.57)	0.067
Immune deficiency	9 (7.96)	7 (8.97)	2 (5.71)	0.718
Acute-on-chronic	9 (7.96)	6 (7.69)	3 (8.57)	1
Trauma	21 (10.62)	8 (10.26)	4 (11.43)	1
Others	14 (12.39)	9 (11.54)	5 (14.29)	0.76
Treatment				
ECMO, n (%)	3 (2.65)	1 (1.28)	2 (5.71)	0.226
Steroids, n (%)	69 (61.06)	43 (55.13)	26 (74.29)	0.053
NMBA, n (%)	35 (30.97)	20 (25.64)	15 (42.86)	0.067
Vasodilator, n (%)	41 (36.28)	28 (35.90)	13 (37.14)	0.899
Plasma inflammatory markers				
TNF-α (pg/mL)	13.23 (9.25–26.85)	12.52 (8.69–15.35)	18.32 (15.69–23.81)	0.04
IL-1β (pg/mL)	5.25 (3.86–7.98)	6.52 (4.32–8.42)	3.05 (2.86–4.62)	0.02
IL-6 (pg/mL)	810 (789–1753)	862 (698–1352)	1425 (1253–1832)	0.04
IL-8 (pg/mL)	8.65 (6.68–13.38)	6.53 (5.21–9.76)	12.31 (10.35–17.60)	0.01
IL-17A (pg/mL)	1.23 (1.02–2.12)	1.15(0.89–1.42)	1.85 (1.68–2.32)	0.01

### Plasma sICAM-1 and sP-Selectins levels in ARDS patients

**Figure 1:**
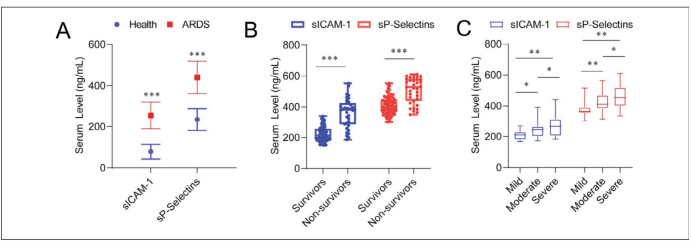
Plasma sICAM-1 and sP-Selectins levels in (A) healthy people (n = 36) and ARDS patients (n = 113); (B) ARDS survivors (n = 78) and non-survivors (n = 35); (C) Mild (n = 11), moderate (n = 50), severe (n = 52) ARDS patients. Data were expressed as median (IQR), and the Mann-Whitney U or Kruskal-Wallis H test compared the differences. *** p < 0.001; ** p < 0.01; * p < 0.05.

**Figure 2:**
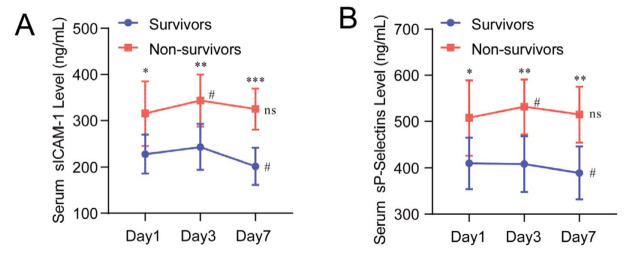
Average plasma concentrations of (A) sICAM-1 and (B) sP-Selectins over time in survivors (n=78) and non-survivors (n=35) of ARDS. Data were expressed as x ± SD. Mann-Whitney U or Kruskal-Wallis H tests compared the differences between groups of independent samples, *** p < 0.001; ** p < 0.01; * p <0.05. Wilcoxon Signed Rank or Friedman M test compared the differences between the paired samples, # p < 0.05.

Plasma sICAM-1 and sP-Selectins levels in ARDS patients were significantly higher than those in healthy controls (*p* < 0.001) (Figure 1A), and in nonsurvivors than in ARDS survivors (*p* < 0.001) (Figure 1B). ARDS patients were classified as mild, moderate, and severe. Plasma sICAM-1 and sP-Selectins levels increased significantly with the severity of ARDS (I < 0.05) (Figure 1C). Nonparametric longitudinal analysis showed significant differences in plasma sICAM-1 and sP-Selectins levels between the two groups at day 1, 3, and 7 of admission, respectively (survivors vs non-survivors; *p* < 0.05, Figure 2). Nonparametric transverse analysis showed that plasma sICAM-1 and sP-Selectins levels increased significantly in non-survivors on day 3 of admission (Day 3 vs. Day 1; *p* < 0.05). However, after day 7, the levels of both factors dropped to the levels of day 1. In addition, plasma sICAM-1 and sP-Selectins levels were not found to increase with time in survivors, but decreased significantly on day 7 (Day 7 vs. Day 1; *p* < 0.05).

### Relationship between plasma sICAM-1, sP-Selectins, and inflammatory factors

Among inflammatory markers, there was no correlation between IL-6 and IL-17A, and a significant correlation was found among other factors to varying degrees. There was a strong positive correlation between IL-6 and IL-8 (0.623, *p* < 0.001). In addition, sICAM-1 had a weak correlation with TNF-α (rs = 0.392, *p* = 0.023), IL-6 (rs = 0.375, *p* < 0.001), and a moderately significant correlation with IL-8 (rs = 0.450, *p* < 0.001). sP-Selectins were weakly correlated with IL-6 (rs = 0.367, *p* < 0.001) and IL-8 (rs = 0.375, *p* = 0.013). Furthermore, a weak and significant correlation was presented between sICAM-1 and sP-Selectins (rs = 0.328, *p* < 0.001). Table 2

**Table 2:** The relationship between plasma sICAM-1, sP-Selectins and inflammatory factors at admission. Spearman’s correlation coefficient was used to analyze the association between the factors, and all Spearman pairs with significant comparison were analyzed with False Discovery Rate to correct p values <0.05.

	TNF-α	IL-1β	IL-6	IL-8	IL-17A	sICAM-1	sP-Selectins
TNF-α	/						
IL-1β	0.565	/					
	(p<0.001)						
IL-6	0.378	0.598	/				
	(p<0.001)	(p<0.001)					
IL-8	0.523	0.384	0.623	/			
	(p<0.001)	(p=0.012)	(p<0.001)				
IL-17A	0.589	0.623	0.435	0.627	/		
	(p<0.001)	(p=0.506)	(p=0.024)	(p=0.058)			
sICAM-1	0.392	0.438	0.375	0.45	0.632	/	
	(p=0.023)	(p=0.241)	(p<0.001)	(p<0.001)	(p=0.182)		
sP-Selectins	0.452	0.359	0.367	0.375	0.588	0.328	/
	(p=0.053)	(p=0.438)	(p<0.001)	(p=0.013)	(p=0.768)	(p<0.001)	

### Prediction values of plasma sICAM-1 and sP-Selectins

**Figure 3:**
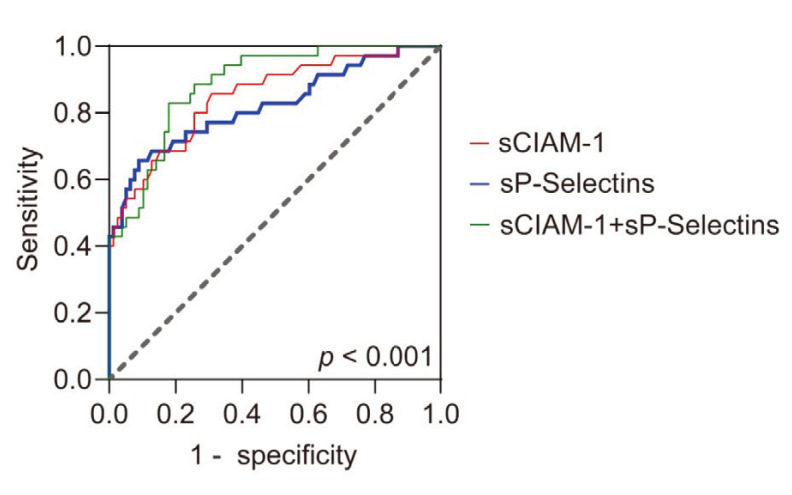
ROC evaluated plasma sICAM-1, sP-Selectins, and their combined measurements to distinguish survival from death.

**Figure 4:**
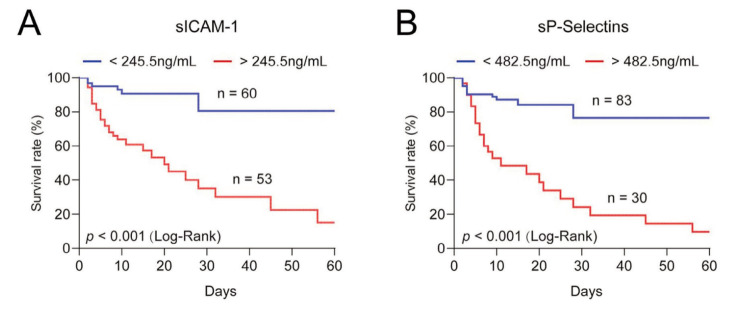
Survival probability of ARDS patients grouped according to plasma critical levels of (A) sICAM-1 and (B) sP-Selectins according to ROC analysis is shown in Kaplan-Meier curve. Logarithmic rank test: p < 0.001.

ROC curves were plotted to further evaluate the predictive quality of plasma sICAM-1 and sP-Selectins in ARDS (Figure 3) and a critical value was calculated based on the Youden index. The threshold that best distinguishes between survival and death was 245.5 ng/mL for plasma sICAM-1 level (AUC = 0.850; 95%CI = 0.770-0.930; *p* < 0.0001; Sensitivity = 85.7%, specificity = 69.2%) and 482.5 ng/mL for plasma sP-Selectins (AUC = 0.822; 95%CI = 0.730-0.914; *P* < 0.0001; Sensitivity = 65.7%, specificity = 91.0%). The death rate of ARDS was 25.67% (29/113) and 20.35% (23/113) in patients with plasma sICAM-1 and sP-Selectins levels above the critical value. The combination of the two had higher predictive value (AUC = 0.886; 95%CI = 0.825-0.948; *P* < 0.0001; Sensitivity = 82.9%, specificity = 82.1%). Univariate binary logistic regression analysis was used to account for potential confounders that influenced death in the analysis. Plasma sICAM-1 (OR = 4.36; 95%CI = 1.89-7.63; *p* < 0.001), sP-Selectins (OR = 2.89; 95%CI = 1.36-4.06; *p* < 0.001) were risk factors for patients' death. In addition, age, yes/no severe ARDS, ICU admission score (SAPS II and APACHE II), and pulmonary compliance were identified as risk factors for death (Table 3). In contrast, gender, BMI, duration of mechanical ventilation, and length of ICU stay were not predictors of death (Table 3). The cumulative survival rate was represented by Kaplan-Meier curve, and the median survival time of patients with sICAM-1 higher than this level was 18 days (log-rank *p* < 0.001; Figure 4A). The median survival time of patients with sP-Selectins above this level was 11 days (log-rank *p* < 0.001; Figure 4B). To further evaluate the critical values of sICAM-1 and sP-Selectins as independent risk factors for predicting death and the critical values of sICAM-1 and sP-Selectins as independent risk factors for predicting death, multivariate stepwise Logistic regression was used for analysis. Factors that were significant in univariate analysis were considered, including age, yes/no severe ARDS, ICU admission score (SAPS II and APACHE II), and pulmonary compliance. When this threshold was exceeded, the risk of death increased (sICAM-1 > 245.5 ng/mL, OR = 3.66, 95%CI = 1.93-5.89, *p* < 0.001; sP-Selectins > 482.5 ng/mL, OR = 2.54, 95%CI = 1.25-3.63, *p* < 0.0001). In addition, APACHE II and pulmonary compliance were identified as independent risk factors for death in ARDS patients (Table 4). In multivariate Cox regression analysis, sICAM-1 and sP-Selectins cutoff values, APACHE II, and pulmonary compliance were independent risk factors for death in ARDS patients (Table 4).

**Table 3:** Univariate Logistic regression analysis of factors affecting death.

Univariate logistic regression analysis	OR	95%CI	p value
Age (years)	1.36	0.63–2.36	0.038
Gender	1.83	0.26–3.32	0.785
BMI	1.36	0.89–2.36	0.412
Severe ARDS (yes/not)	1.63	1.07–3.48	0.036
SAPS II (admission)	2.36	0.98–4.36	0.053
APACHE II (admission)	5.69	2.32–9.64	< 0.001
CL (admission)	0.862	0.36–0.99	0.04
Mechanical ventilation (admission)	0.369	0.23–1.35	0.06
ICU length of stay (days)	2.38	1.36–5.36	0.046
sICAM-1 (admission)	4.36	1.89–7.63	< 0.001
245.5 ng/mL			
sP-Selectins (admission)	2.89	1.36–4.06	< 0.001
482.5 ng/mL			

**Table 4:** Logistic regression and Cox risk regression analysis of multiple factors influencing death. Multivariate logistic regression model and Cox proportional risk regression model were used for analysis. OR, odds ratio; CI, confidence interval.

Multivariate logistic regression	OR	95%CI	p value
APACHE II (admission)	6.32	3.36–10.52	< 0.001
pulmonary compliance (admission)	0.76	0.63–0.98	0.032
sICAM-1 (admission)	3.66	1.93–5.89	< 0.001
245.5 ng/mL			
sP-Selectins (admission)	2.54	1.25–3.63	< 0.001
482.5 ng/mL			
Cox regression	HR	95%CI	p value
APACHE II (admission)	2.31	1.35–2.63	< 0.001
pulmonary compliance (admission)	0.97	0.96–0.99	0.048
sICAM-1 (admission)	1.56	1.02–1.86	< 0.001
245.5 ng/mL			
sP-Selectins (admission)	1.25	1.05–1.63	< 0.001
482.5 ng/mL			

## Discussion

ARDS is a multifactor life-threatening lung injury characterized by diffuse pneumonia and increased permeability of alveolar capillary barrier [23]. Our study found that sICAM-1 and sP-Selectins at admission were correlated with death, and exceeding the threshold of sICAM-1 and sP-Selectins can effectively predict the risk of death in ARDS patients, and the combined prediction of the two has a higher evaluation value. There was a significant correlation between sICAM-1 and sP-Selectins and different inflammatory factors. Inaddition, admission APACHE II score and pulmonary compliance were established as independent risk factors for death in ARDS patients.

To the best of our knowledge, this is the first study to determine the correlation between plasma sICAM-1 and sP-Selectins levels and their combination in predicting the risk of death in adults with ARDS. The population in this study was characterized by severe illness, which is reflected in high admission scores for APACHE II and SOFA II. Patients were classified into mild, moderate, and severe ARDS according to the Berlin definition. As most ARDS cases develop rapidly within minutes to hours, rather than days [24], this study observed more patients with moderate and severe ARDS. Mechanical ventilation is a therapeutic means to prevent secondary lung injury and improve patient prognosis in ARDS management. As expected, non-surviving patients were more likely to develop severe ARDS and showed significantly lower lung compliance, longer periods of mechanical ventilation, and longer ICU stays. However, no difference was found in PEEP between survivors and non-survivors, although PEEP is commonly used in patients with severe ARDS [25]. Our study identified higher plasma sICAM-1 and sP-Selectins levels in ARDS patients and higher levels in non-survivors of ARDS. In a larger ARDS study, a significant increase in IL-6 has been found, and both IL-6 and IL-8 are associated with worse outcomes [26]. In our study cohort, non-survivors were strongly associated with higher plasma TNF-α, IL-1β, IL-6, IL-8, and IL-17A, and there was asignificant and strong positive correlation between IL-6 and IL-8. However, IL-6 had no significant correlation with IL-17A, although IL-6 has been reported to induce pathogenic Th17 cells through the trans-presentation of dendritic cells [27]. The relationship between endothelial cell markers sICAM-1 and sP-Selectins and inflammatory factors in ARDS patients is not completely clear. However, systemic or local inflammation with high levels of proinflammatory cytokines is considered to be an effective trigger for endothelial factor activation and release into the peripheral circulation [28] [29]. Studies have confirmed that IL-1β and TNF-α significantly enhance the expression or release of ICAM-1 and P-Selectins [30] [31]. In our study, sICAM-1 was significantly correlated with TNF-α, IL-6 and IL-8, and sP-Selectins was also significantly correlated with IL-6 and IL-8, while no correlation was found with IL-17A.

Considering the rapid progression of ARDS, plasma sICAM-1 and sP-Selectins may change with time. In addition to the day of admission (day 1), plasma sICAM-1 and sP-Selectins were measured on day 3 and day 7. The results showed that plasma sICAM-1 and sP-Selectins levels in non-survivors increased significantly at day 3, although this study did not perform continuous measurements to obtain complete trends in the cohort study. However, it is worth noting that on day 7, plasma sICAM-1 and sP-Selectins both decreased to similar levels as on day 1. Reliable biomarkers may be useful both in identifying patients at risk of developing ARDS and in monitoring treatment progress in the ICU. Further prospective clinical studiesare needed to determine whether plasma sICAM-1 and sP-Selectins can be used as biomarkers for ARDS. 

Furthermore, plasma sICAM-1 and sP-Selectins were independently associated with a higher risk of death. This is consistent with some previous reports that sICAM-1 and sP-Selectins detection are associated with relatively poor prognosis of some diseases [32] [33]. However, in our study, some patients below the sICAM-1 and sP-Selectins cutoff values survived, while some above the cutoff value died, and vice versa. Nevertheless, high plasma sICAM-1 and sP-Selectins at ICU admission were independent predictors of death in ARDS patients and may have a high prognostic ability. Furthermore, our analysis showed that cutoff values of 245.5 ng/mL (sICAM-1) and 482.5 ng/mL (sP-Selectins) were the most appropriate levels to distinguish between survival and death, yielding the highest sensitivity and specificity values. When this threshold is exceeded, the death rate in people with ARDS increases by more than two to four times. Given the high death risk inherent in ARDS, a more than two-fold increase in the risk of death has considerable implications [2]. Interestingly, in this study, sICAM-1 and sP-Selectins were not only independent predictors of death, but also showed on the ROC curve that sICAM-1 combined with sP-Selectins analysis had greater diagnostic value. This further supports the view that sICAM-1 and sP-Selectins can be used as accurate markers of ARDS death risk. In addition, sICAM-1 has been identified as a strong predictor of in-hospital death in critically ill pediatric ARDS patients [34]. Although this study did not analyze whether sICAM-1 combined with sP-Selectins was an independent risk factor for death, these data suggest that sICAM-1 and sP-Selectins may be strong predictors of outcome in critically ill patients even after discharge from the ICU.

The study is a prospective pooled cohort designed specifically to assess risk factors and outcomes for ARDS, a clear population with detailed descriptions of ARDS etiology and covariates, and adequate adjustment for important baseline features. However, considering that this study was only conducted in Qingpu Branch of Zhongshan Hospital Affiliated to Fudan University, the size of included subjects was small and the statistical power was limited. Future larger studies could adjust for multi-variables and identify potential interrelationships or risk factors not identified in current work. More effective use of this parameter as a marker for predicting the risk of ARDS death provides important information.

## Dodatak

### Acknowledgments

Not applicable.

### Funding

Not applicable.

### Availability of data and materials

The datasets used and/or analyzed during the present study are available from the corresponding author on reasonable request.

### Ethics approval and consent to participate

The present study was approved by the Ethics Committee of Qingpu Branch of Zhongshan Hospital Affiliated to Fudan University and written informed consent was provided by all patients prior to the study start. All procedures were performed in accordance with the ethical standards of the Institutional Review Board and The Declaration of Helsinki, and its later amendments or comparable ethical standards.

### Conflict of interest statement

All the authors declare that they have no conflict of interest in this work.
